# Adoption of digital tools in the context of the COVID-19 pandemic in the Region of the Americas - the Go.Data experience

**DOI:** 10.1016/j.lana.2022.100377

**Published:** 2022-10-06

**Authors:** Cristina Valencia, Giovanna Jaramillo-Gutierrez, Analía Rearte, Paula Rosin, Fernando Gassino, Silvia Edith Morreale, Lorena Gobern, Antonio Paredes, Marc Rondy, Evelyn Balsells, Pablo Galindo, Liz Parra, Oliver Mazariegos, Amy Young, Darlene Bhavnani, Aaron Miri, Daniel Iken, Emily James, Angel Rodriguez

**Affiliations:** a Global Outbreak Alert and Response Network (GOARN), Geneva, Switzerland; b Department of Epidemiology, Mailman School of Public Health, Columbia University, New York City, USA; c European Programme of Interventional Epidemiology Training Alumni Network, EAN, Brussels, Belgium; d Department of Epidemiology, Ministry of Health, Buenos Aires, Argentina; e National University of Mar del Plata, Medicine School, Buenos Aires, Argentina; f Department of Information Systems, Ministry of Health, Buenos Aires, Argentina; g Department of Health Emergencies, Pan American Health Organization, Buenos Aires, Argentina; h Department of Epidemiology, Ministry of Health, Guatemala City, Guatemala; i Immunization Unit, Pan American Health Organization, Guatemala City, Guatemala; j Usher Institute of Population Health Sciences and Informatics, University of Edinburgh, Medical School, Teviot Place, Edinburgh, United Kingdom; k Department of Health Emergencies, Pan American Health Organization, Guatemala City, Guatemala; l The Dell Medical School at University of Texas at Austin, UT Health Austin, Austin, USA; m Department of Health Emergencies, Pan American Health Organization, Washington, United States of America

**Keywords:** Region of the Americas, Digital health, COVID-19, SARS-CoV-2, Surveillaince, Outbreak response, Contact tracing, Digital tools, Go.Data, Interoperability

## Abstract

The COVID-19 pandemic has accelerated the growth of digital health tools. Although a number of different tools exist to support field data collection in the context of outbreak response, they have not been sufficient. This prompted the World Health Organization (WHO) to collaborate with the Global Outbreak Alert and Response Network (GOARN) and GOARN partners to develop a comprehensive system, Go.Data. Go.Data, a digital tool for outbreak response has simplified how countries operationalize and monitor case and contact data. Since the start of the pandemic, WHO and GOARN partners have provided support to Go.Data projects in 65 countries and territories, yet the demand by countries to have documented success cases of Go.Data implementations continues to grow. This viewpoint documents the successful Go.Data implementation frameworks in two countries, Argentina and Guatemala and an academic institution, the University of Texas at Austin.

## Introduction

On 30 January 2020, the World Health Organization (WHO) declared the COVID-19 outbreak a public health emergency of international concern.^1^ The first case in the Americas was confirmed in the United States on 20 January 2020, followed by Brazil on 26 February, 2020.^2^ Since then, COVID-19 has spread to all 54 countries and territories in the Americas. As health systems in the region were placed under severe pressure, governments and public health authorities needed to find innovative digital technologies to help effectively and efficiently contain the pandemic.

The rapid expansion of digital health made the powerful role new technologies play in overcoming common challenges faced in outbreak response evident.^3^ Building on lessons learned in responding to past emergencies, and identifying data management as a critical pillar in pandemic response,^4^ WHO collaborated with partners from the Global Outbreak Alert and Response Network (GOARN) to develop Go.Data.

Go.Data is an outbreak investigation tool for field data collection. Field-based users such as epidemiologists, contact tracers, and laboratory staff can register cases, contacts, and their related data.^5^ Since the start of the pandemic, WHO and GOARN partners have supported Go.Data projects in over 65 countries or territories, where implementations have been conducted at national, sub-national and institutional level. In the Americas, 31 countries and territories have been trained and 18 continue to actively use Go.Data for outbreak response activities.^6^

Go.Data has strengthened the systematization, communication, and notification of case and contact data including the management and integration of information at different levels within health systems. Yet, challenges around data volume, privacy, human resources, logistics, financing, and the numerous digital tools already available presented issues for many countries.

In this viewpoint, we describe the experiences, challenges, and best practices of the adoption and implementation of Go.Data in three use cases in the Americas: Argentina, Guatemala and the University of Texas at Austin, to demonstrate how it has shaped outbreak response for infectious disease threats.

## Go.Data

Go.Data is a custom-built software developed by WHO in collaboration with GOARN partners to support data collection during outbreak response, including the management of case and contact data. It consists of a web application (for Windows, Linux and Mac) which can run either as a standalone or server installation, and an optional mobile phone application available for both iOS and Android. The mobile phone application works only in conjunction with the web application. The web application consists of a NoSQL database (MongoDb), and the front-end utilizes NodeJS, Angular and open source components which are used to deliver all required features and functionalities. Go.Data contains a dashboard with essential indicators, additional dashboards can be created using third-party platforms and Go.Data Application Programming Interface (API).

## Use cases in practice

### Argentina

Argentina, with a population of 45.8 million is located in the southern half of South America. Its provinces and capital exist under a federal system.^7^ During the last two decades, Argentina has worked to strengthen epidemiological surveillance through different national strategies, one of them being the development of the national health surveillance system called *Sistema Nacional de Vigilancia de la Salud* (SNVS^2.0^).^8^^,^^9^ The implementation of the SNVS^2.0^ by the Ministry of Health (MoH) allowed the country to strengthen the registration and monitoring of notifiable diseases at the population level.

The SNVS^2.0^ facilitates the notification of compulsory events and the systematic and rapid analysis of data from clinical and laboratory surveillance, sentinel units, and disease-specific programs. The SNVS^2.0^ user network is comprised of over 18 thousand active users distributed throughout the country.

At the beginning of 2020, the COVID-19 pandemic prompted the need to expand the capacities of SNVS^2.0^. Given the lack of available digital tools to support outbreak investigation, the MoH defined the implementation of Go.Data as an integrated module of the SNVS^2.0^ which was part of their broader strategic plan to enhance epidemiological surveillance and response. The objective was to provide a tool for jurisdictions to facilitate epidemiological investigations and contact tracing based on data stored in the SNVS^2.0^.

**Figure 1:**
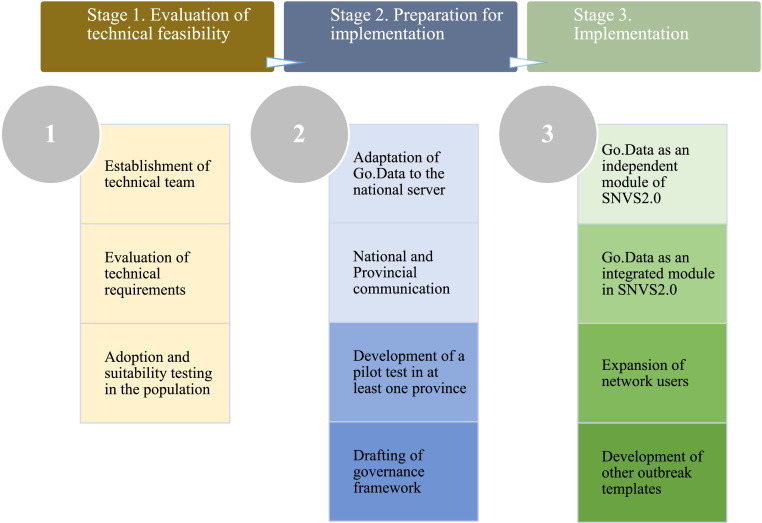
**Structural framework of the staged implementation of Go.Data by the Ministry of Health in the context of the COVID-19 pandemic, Argentina, April 2020- October 2021**. SNVS denotes Sistema Nacional de Vigilancia de la Salud.

The rollout of Go.Data within the SNVS^2.0^ framework was carried out through gradual and systematic implementation stages targeting the 24 jurisdictions from April 2020 to October 2021 (Figure 1). These included 1) Evaluation of the technical feasibility 2) Preparation for implementation and 3) Implementation.

During the first stage, a national Go.Data multidisciplinary team was established to evaluate the technical requirements and suitability of the tool. The team was composed of two pillars: 1) the epidemiology pillar, in charge of training, management, platform configuration, and development of training materials 2) the information systems pillar, in charge of installing Go.Data, establishing system interoperability and maintenance.

**Figure 2:**
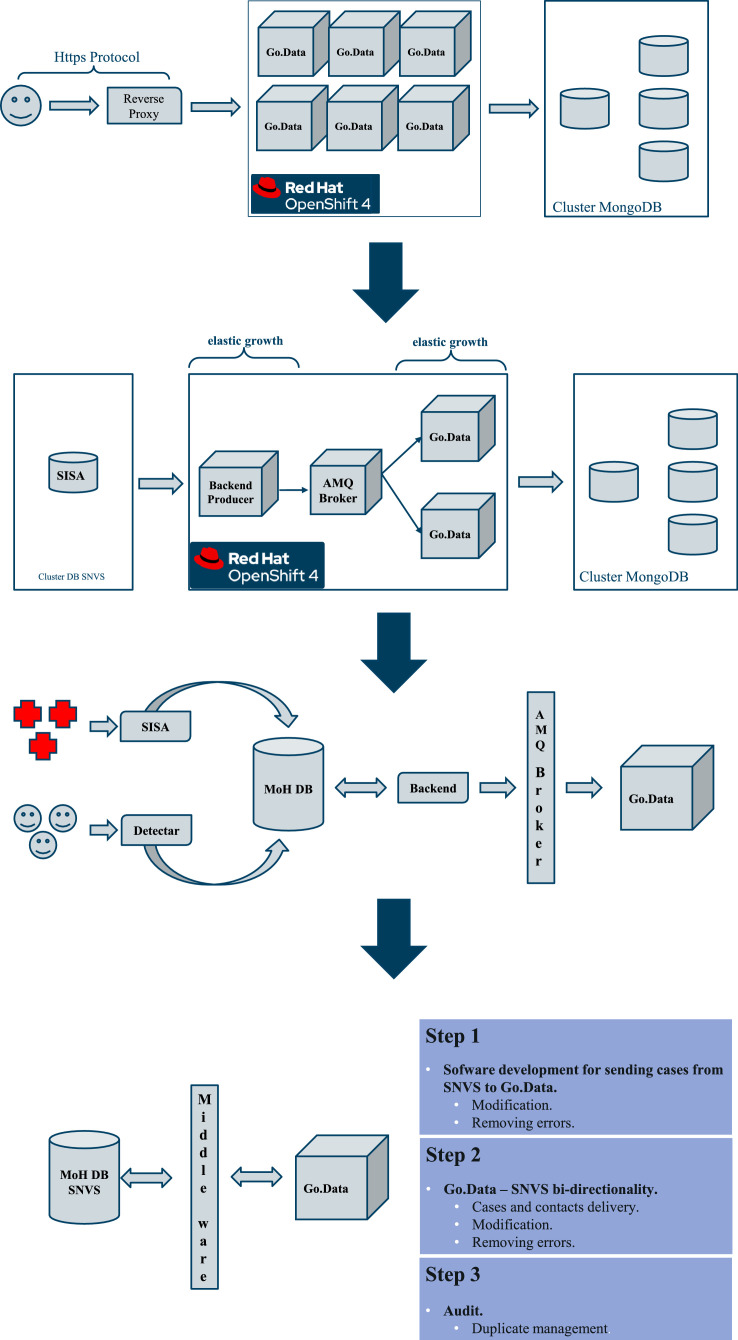
**Structure of Go.Data installation using a container orchestration system, including software architecture, data flow and interoperability structure with the SNVS (National Surveillance System) Argentina, April 2020- October 2021**. AMQ denotes advanced message queuing. DB denotes database. MoH denotes ministry of health. SISA denotes Sistema Integrado de Información Sanitaria Argentino. SNVS denotes Sistema Nacional de Vigilancia de la Salud.

In stage 2, Go.Data was installed in a container orchestration system (Kubernetes) on an OpenShift platform due to the high volume of data that the national systems were already receiving (Figure 2). In parallel, a communication campaign was launched which included capacity building through the development of country-specific training tools.^10^ At the end of this stage, a pilot project was conducted in one of the provinces and the Go.Data governance framework was drafted.

In the last, and still ongoing stage, Go.Data was integrated into the SNVS^2.0^. The interoperability between SNVS^2.0^ and Go.Data was managed by a middleware which aims to establish a bi-directional connection of information. The architecture of the interaction between these two systems is outlined in Figure 2.

During the implementation period, almost 800 health workers from 22 provinces were trained on the use of Go.Data and epidemiological dashboards were generated using Power BI, connected to Go.Data and shared with each jurisdiction to facilitate data analysis.

Challenges were identified throughout the implementation of Go.Data. First, local teams felt overwhelmed in their capacity to respond to the evolving pandemic and fears about implementing new technologies. Second, the multiplicity of digital tools available for use. Finally, the interoperability between SNVS^2.0^ and Go.Data given the different logics and programming languages of each system.

The implementation of Go.Data in Argentina made it possible for the MoH to provide a tool to jurisdictions that facilitated monitoring and analyzing chains of transmission while strengthening contact tracing activities. It responded to user demands by supporting outbreak investigations and improving systematization, communication, notification and integration of information at different health management levels. Go.Data continues to be used in Argentina and strategies for its future use with other infectious diseases such as monkeypox, hantavirus, tuberculosis, and measles are under development.

### Guatemala

Guatemala is a country in Central America with a population of 17 million. ^11^ Its MoH is organized into local authorities (29 health areas, 46 hospitals) and a central office, from where the Epidemiology Department conducts normative activities and provides technical support and disease surveillance monitoring. The notification and investigation of epidemiological events are carried out by the local authorities and reported to the central level. Multidisciplinary rapid response teams are trained at the local level to respond to outbreak potential events.

The first COVID-19 case was detected in Guatemala on March 13, 2020.^12^ By then, the MoH had developed COVID-19 surveillance guidelines, including case detection and investigation and contact tracing strategies.^13^ In the early stage of the pandemic, case investigation and contact follow-up were monitored using excel files. This methodology quickly became challenging for healthcare workers to maintain. Go.Data was presented by the Pan American Health Organization (PAHO) to the Epidemiology Department on April 24, 2020. Between May and November 2020, the MoH implemented Go.Data to facilitate case isolation follow-up, contact listing and quarantine follow-up. During the first stage of Go.Data implementation (May-July 2020), the tool was installed in a centralized server using a Windows platform. The MoH Epidemiology Department developed and distributed a technical document outlining the process of data flow. Questionnaires generated in Go.Data were used for case and contact follow up and the excel-stored historical data were imported into the system. The implementation team focused on capacity building through virtual trainings of 630 participants. In parallel, a country-specific user manual and short instructional videos were developed in Spanish and published on the MoH website. During this phase, 20 (69%) health areas continuously used the platform.

**Figure 3:**
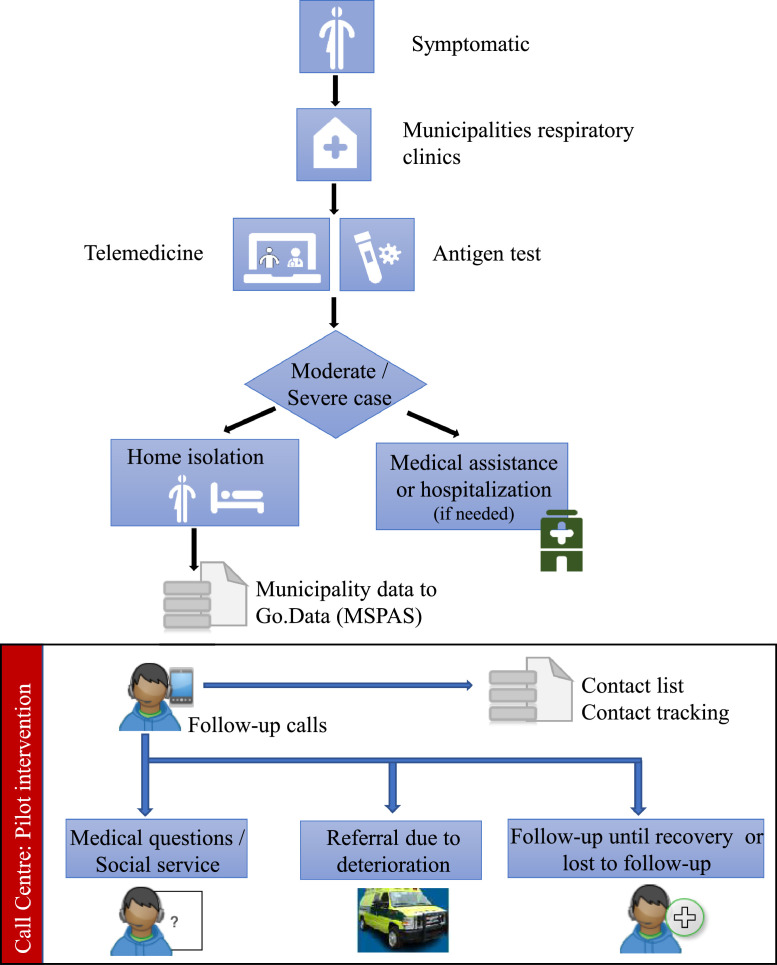
**Information flow for COVID-19 case and contact follow-up from symptom onset to follow-up and recovery, Guatemala, May 2020- November 2020**. MSPAS denotes Ministerio de Salud Pública y Asistencia Social.

Following this first phase, a pilot project was conducted in Guatemala City to integrate case detection with remote case and contact follow-up (August-November 2020). A multidisciplinary team, including staff from the Guatemala City Health Board and Municipality and the support of international agencies, was built under the coordination of the MoH.^14^ The project consisted of one coordinator (epidemiologist), four supervisors (three with clinical background), 55 contact tracers, five data contact tracing teams managers, and one bioinformatician/programmer. Figure 3 shows the case and contact follow-up system and information flow. Questionnaires in Go.Data for cases were customized so that results from follow-up calls could be recorded and data analyzed. Training material on COVID-19 recommendations and Go.Data was developed for newly hired contact tracers. To support decision-making and monitoring of the intervention, daily reports were dispatched by email to decision makers (at the central level) and a virtual dashboard in R Shiny was developed. The dashboard processed data directly from Go.Data API's and provided visualizations and indicator analysis needed to address the questions coming from decision makers at weekly meetings.

Scaling up the pilot project for call-based contact tracing presented technical and context-specific challenges such as limited internet connectivity, human resources for contact tracing and management, inadequate accessibility to technology to conduct remote follow-up (laptop computers and mobile phones with the necessary data plans), and lack of trust to disclose personal information over the phone (due to high levels of surveillance insecurity and extortion). Overcoming these challenges was only possible to a certain extent due to the resources available for the project. Go.Data is still used in Guatemala City. The MoH Epidemiology Department is planning to use Go.Data nationally utilizing the lessons learned from the experience in the capital city, mostly based on remote follow-up given the limited resources available for home-based contact tracing (personal protective equipment, transport) and insecurity issues.

### University of Texas Austin

The University of Texas at Austin (UT), with approximately 51 thousand students enrolled each year, is one of the largest public universities in the United States. On March 13, 2020, the first COVID-19 case was reported at the University of Texas Health Austin (UTHA), the clinical practice of the Dell Medical School. UTHA collaborated with other health entities on campus to establish a consortium for COVID-19 management, which included a contact tracing workforce comprised of medical students. The consortium led the campus’ initial response and pandemic management with contact tracing conducted under the authority granted by Austin Public Health. COVID-19 pandemic management included testing, mental health management, vaccination, and therapeutic administration. Data sharing between entities was essential for cluster investigations, notification, and testing.

Testing centres were opened within UTHA and University Health Services following the identification of COVID-19 cases linked to a spring break trip on March 27, 2020, and contact tracing activities expanded to include an array of multidisciplinary teams. Response operations were rapid, but the necessary data systems took longer to evolve. As data volumes grew, the need for a scalable enterprise solution became evident. After carefully evaluating different platforms for outbreak response, the UTHA team selected Go.Data for its flexible architecture and capacity to integrate into existing data systems. Within three weeks, operational processes, which included data collection and data management, were established.

**Figure 4:**
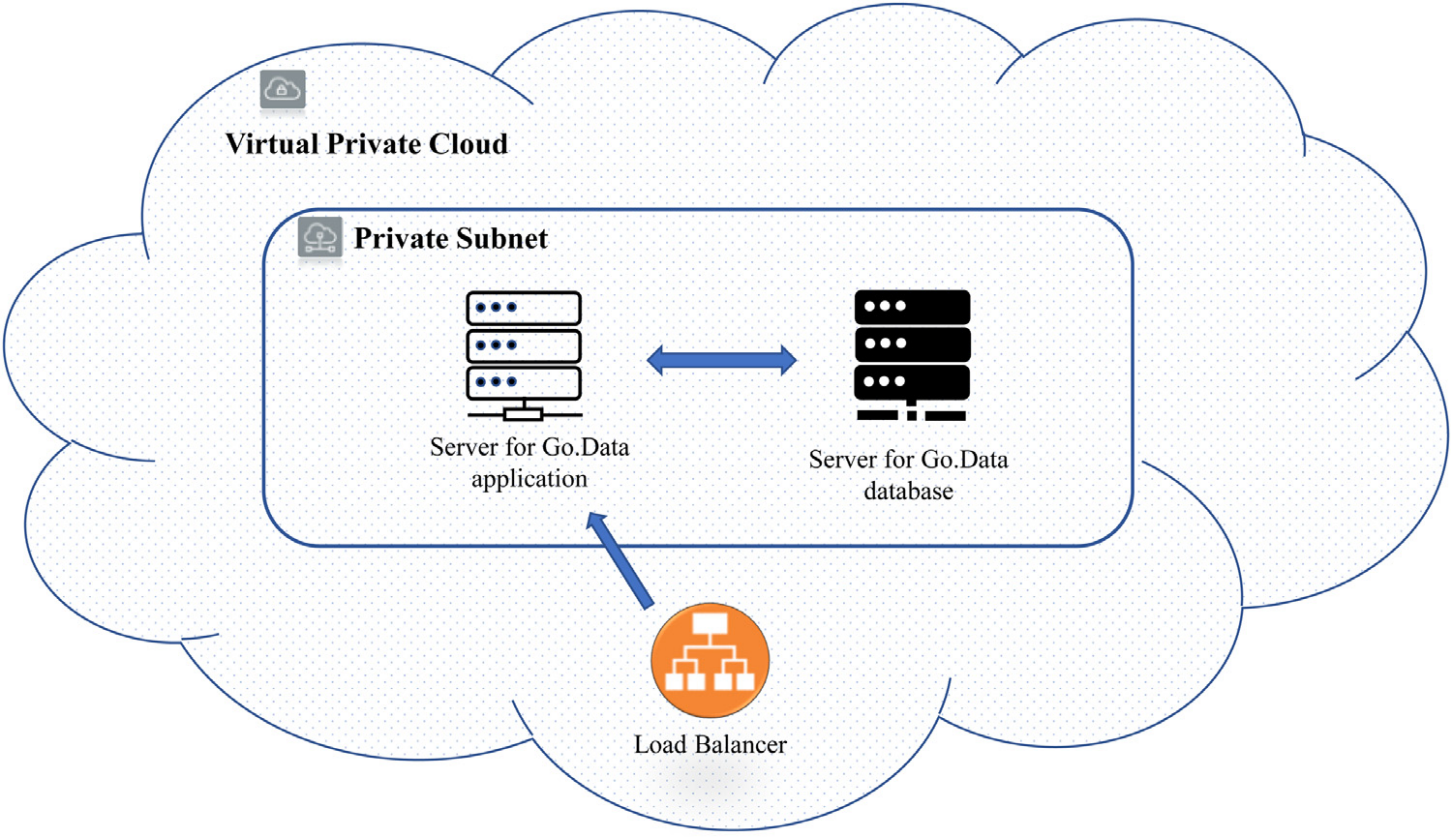
**Structure of Go.Data installation through a distributed server configuration, including software architecture, University of Texas Austin, August 2020- May 2022**.

Installation of Go.Data was done through a distributed server configuration for scalability and stored on Amazon web services for cybersecurity compliance, as seen in Figure 4. The server where Go.Data was installed was scaled to six virtual servers using a load balancer, while the Go.Data database remained in a standalone server situated within a virtual private cloud and a subnet.

This scalable cloud infrastructure allowed UTHA contact tracing team members to use Go.Data remotely by logging into the platform through a two-factor authentication process.

Strategies for the recruitment, training and performance management of the contact tracers were also developed, as was a comprehensive online training platform that enabled capacity building. Through a hierarchical management structure, contact tracers were supervised by team leads who ensured that all data was collected and the toolwas used correctly. Go.Data provided team leads with operational performance metrics as well as innovative ways of monitoring contacts. Between August 2020 and May 2022, UT Austin relied on 18 staff members and 150 volunteers to trace the contacts of 10,731 cases managed in Go.Data.

Indicators in the Go.Data dashboards were insufficient to inform non-technical audiences, which led to the development of more detailed dashboards using Tableau. These dashboards included COVID-19 coverage and impact, testing and tracing delays, and exposure sites amongst others. Data feedback loops between the dashboards and Go.Data allowed for more efficient analyses, for example, sequencing of all confirmed COVID-19 cases led the team to incorporate variant-level details in the platform so that visualizations of chains of transmission could detect variant linkage. Go.Data, also facilitated ad-hoc cluster analyses to detect trends by building, student organization, athletic teams, and housing zones.

While not required for contact tracing, Go.Data allowed teams to collect, analyze, visualize, and report their data. An example of this was the expansion of testing to the West campus given the development of clusters that were being identified through Go.Data. This highlighted the value of the platform within that context.

Given the congregate nature of a university setting, Go.Data facilitated the management and coordination of campus-based contact tracing. Challenges, however, included a high turnover of contact tracers, budget, platform updates, administrative approvals, security audits, and data sharing agreements. Despite this, Go.Data should be considered for future outbreak response management.

## Lessons learned and recommendations for future implementation

Examination of Go.Data implementations across these three use cases reveal several commonalities and shared challenges (Table 1). We highlight the key lessons learned for future implementations.

**Table 1:** Comparison of Go.Data implementation processes^a^ between the three use-cases; Argentina, Guatemala and University of Texas at Austin, 2020-2022.

	Server Configuration	Analytics Plug-in	Training Modality	Implementation period	Active Users	Number of cases	Interoperability
**Argentina**	Container Orchestration system (Kubernetes)		Training modules on MoH website; Provincial communication campaigns	April 2020 October 2021	800	85,830	SNVS^2.0^ / SISA^b^
**Guatemala**	Centralized server at MoH Epidemiology Department		Virtual training workshops with local response teams; follow-up in-person refreshers	May 2020 – November 2020	65	12,953	Epiweb national epidemiology center
**University of Texas at Austin**	Distributed cloud (AWS^b^) within VPC^b^		Online curriculum delivered by Training Coordinator and Team leads	August 2020 – May 2022	··	10,731	APH^b^ database

a Including server configuration, analytics, training modalities, number of active users and interoperability with other systems.

b APH database denotes Actual Production History. AWS denotes Amazon Web Services. MoH denotes Ministry of Health. SISA denotes Sistema Integrado de Información Sanitaria Argentino. SNVS^2.0^ denotes Sistema Nacional de Vigilancia de la Salud. VPC denotes Virtual Private Cloud.

### Multi-disciplinary teams

All three use cases implemented Go.Data in the early stages of the pandemic with sizeable and multi-disciplinary teams, which helped tailor the tool to its specific context. Given the challenges faced by having such a team during a period of limited resources, pooling of expertise from existing in-country networks to supplement core teams is recommended.^15^ Multidisciplinary teams should also be tasked with the sustainability of the tool beyond COVID-19.

### Server optimization and IT infrastructure

With the unprecedented scale and magnitude of COVID-19 cases and contact data being collected by existing health systems, Go.Data installations that look beyond a single standalone server should be considered. These may include cloud and containerized architectures, amongst others.

### Strong focus on interoperability and data sharing across entities

Interoperability between health systems and Go.Data must be considered when ensuring sustainable implementation and scale. By allowing data exchange between systems, countries and institutions facilitate case and contact identification and monitoring at different administrative levels. This can be enhanced by the generation of data-sharing agreements between institutions and the MoH.

### Analytics to inform decision making

Connecting Go.Data to a visual analytic platform such as Tableau, Power BI, and R Shiny can aid in generating specific indicators. Through these visualizations, data-driven decision-making across relevant stakeholders can be achieved. This further automates public health reporting, informs field operations, and allows for performance monitoring of contact tracers and activities over time. Visualizations that are accessible, easy to interpret, and can be fed back to field teams should be considered, as this can establish synchronicity and timeliness in the way data and indicators are analyzed and presented to response teams.

### Context-appropriate adaptations

Perspectives from Go.Data end users are essential for any implementation, this will require adjusting the tool to the needs of the country or institution in which it is being implemented.^16^ Adapting Go.Data to comply with country-specific guidelines is recommended, this may include translation of training materials and user interface to the local language, customization of training modules based on local needs, the inclusion of case follow-up data, and iteration of questionnaires and workflows depending on the epidemiological situation.

## Contributors

CV and GJG were responsible for conceptualizing the viewpoint and writing the first draft; AR, PR, FG, SM, LG, AP, MR, EB, PG, LP, OM, DB, AY, DI, AM, EJ, ANR performed critical revisions of the viewpoint and contributed to the writing. All authors reviewed and agreed on the final version of the viewpoint. ANR audited the findings.

## Declaration of interests

All other authors declare no competing interests.

## Appendix Supplementary materials

Image, application 1

Image, application 2
