# Supplementary material for: Adoption of digital tools in the context of the COVID-19 pandemic in the Region of the Americas - the Go.Data experience
Source: Lancet Reg Health Am. 2022 Oct 6;16:100377. doi: 10.1016/j.lana.2022.100377 (PMC9536219; doi:10.1016/j.lana.2022.100377)
Supplement: Supplementary file 1 [file mmc1.docx]

**Supplementary material to “Adoption of digital tools in the context of the COVID-19 pandemic in the Region of the Americas - The Go.Data Experience” by Valencia et al.,**

Contents

1. **SPANISH TRANSLATION OF SUMMARY 2**
2. **GO.DATA WEBSITE AND COUNTRY SPECIFIC RESOURCES 3**

# *Editorial Disclaimer: This translation in Spanish was submitted by the authors and we reproduce it as supplied. It has not been peer reviewed. Our editorial processes have only been applied to the original abstract in English, which should serve as reference for this manuscript.*

# SPANISH TRANSLATION OF SUMMARY

*Resumen*

La pandemia de COVID-19 ha acelerado el desarrollo de herramientas digitales en salud. Si bien existen

diversas herramientas para apoyar el registro de datos en terreno en el contexto de respuesta a brotes, las mismas no han sido suficiente. Esto llevó a que la Organización Mundial de la Salud (OMS) junto con la Red Mundial de Alerta y Respuesta a Brotes Epidémicos (GOARN) y sus socios desarrollaran un sistema integral, Go.Data.

Go.Data, una herramienta digital para la respuesta a brotes epidémicos, ha simplificado la fo rma en que los

países operacionalizan y monitorean los datos de casos y contactos. Desde el comienzo de la pandemia, la OMS y los socios de GOARN han apoyado proyectos de Go.Data en 65 países y territorios, lo que aumentó la

demanda de documentación de casos de implementación exitosos. Este punto de vista documenta los casos de

implementación exitosa de Go.Data en dos países, Argentina y Guatemala, y en una institución académica, la Universidad de Texas en Austin.

# GO.DATA WEBSITE AND COUNTRY SPECIFIC RESOURCES

Go.Data website: <https://www.who.int/tools/godata>

*Argentina*

Ministry of Health Go.Data Page: <https://www.argentina.gob.ar/salud/epidemiologia/go-data> Sistema Nacional de Vigilancia (SNVS2.0): <https://sisa.msal.gov.ar/sisa/#sisa>

*Guatemala*

Ministry of Health Go.Data Page : <https://godataguatemala.mspas.gob.gt/auth/login> Strategy for COVID-19 contact tracing using digital tools:

<http://epidemiologia.mspas.gob.gt/informacion/coronavirus-2019-ncov/rastreo-casos-contactos>
